# A case report of sequential efgartigimod and rituximab treatment for tSNMG

**DOI:** 10.3389/fimmu.2026.1848629

**Published:** 2026-09-03

**Authors:** Hua Xie, Ting Ting Pan, Lin Zhang

**Affiliations:** 1 The Second Clinical Medical College of Guizhou University of Traditional Chinese Medicine, Guiyang, China; 2 Department of Neurology, The Second Affiliated Hospital of Guizhou University of Traditional Chinese Medicine, Guiyang, China; 3 Guizhou University of Traditional Chinese Medicine, Guiyang, Guizhou, China

**Keywords:** case report, efgartigimod, myasthenia gravis, rituximab, triple-seronegative

## Abstract

Trible‐seronegative myasthenia gravis (tSNMG) is defined as myasthenia gravis (MG) without detectable or low affinity antibodies to acetylcholine receptor (AChR), muscle‐specific kinase (MuSK) andlipoprotein related protein 4(LRP-4). This article reports a case of a 39-year-old married female patient with thymoma-associated seronegative myasthenia gravis (tSNMG), which was accompanied by multiple positive autoantibodies and a benign mediastinal lesion. The primary manifestation was generalized muscle weakness, involving the limbs, bulbar muscles, and respiratory muscles. After admission, the patient showed poor responses to treatments including pyridostigmine bromide, human immunoglobulin, glucocorticoids, and tacrolimus. Ultimately, following therapy with efgartigimod followed by rituximab, significant symptomatic improvement was observed. By analyzing the diagnosis and treatment process of this case alongside relevant literature, this report explores the therapeutic value of efgartigimod followed by rituximab for tSNMG, key points for individualized regimen adjustments, and the challenges in diagnosis and treatment, thereby providing a reference for the clinical management of such refractory cases.

## Introduction

tSNMG(Triple seronegative myasthenia gravis)is a neuroimmune disorder characterized by clinical and electrophysiological features consistent with MG but lacking antibodies against AChR, MuSK, and LRP4 (1), accounting for 10–15% of all MG. Currently, standard treatment regimens such as acetylcholinesterase inhibitors, glucocorticoids, and immunomodulatory drugs are recommended for antibody-positive MG but are often extended to antibody-negative patients (2). However, randomized controlled trials (RCTs) evaluating targeted biologics often completely exclude tSNMG patients or include only a very small number of cases, which makes it difficult to accumulate a sufficient sample size, thereby limiting statistical power. Consequently, evidence on the efficacy of FcRn antagonists and monoclonal antibodies in treating tSNMG remains extremely scarce. This study uses a case of tSNMG as a starting point to evaluate the real-world efficacy of sequential treatment with an FcRn inhibitor and rituximab, and to preliminarily explore the diagnostic and therapeutic challenges of tSNMG and potential future treatment pathways.

## Case report

A 39-year-old female patient presented to the neurology outpatient clinic of the authors’ hospital with a chief complaint of ‘generalized fatigue for over one month’. One month prior, the patient developed generalized fatigue, difficulty opening the eyes, blurred vision after prolonged visual effort, masticatory weakness, difficulty lifting both upper limbs, weakness in standing and walking, ability to walk only a short distance, intermittent dyspnea, occasional cough, dyspnea on exertion, chest tightness with a burning sensation, a subjective sensation of a mass in the anterior chest, without fever, dysphagia, choking on liquids, or other discomfort. She had a history of gastritis for over two years. She was 155 cm tall and weighed 50 kg, with no recent weight change. Her motor developmental milestones were normal.

Physical examination: body temperature normal, heart rate 69 beats/min, blood pressure 102/68 mmHg, respiratory rate 20 breaths/min, oxygen saturation 95%. The remainder of the cardiopulmonary and abdominal examination was unremarkable. Neurological examination: neck flexion and head lifting for 10 seconds; right upper limb held outstretched for 16 seconds, left upper limb for 15 seconds; left lower limb straight leg raise for 6 seconds, right lower limb for 7 seconds; right hand grip strength 12.7 kg, left hand grip strength 8 kg. When counting from 1 to 32, the patient exhibited effort and slurred speech. On lateral gaze to the left and right, blurred vision (diplopia) developed at approximately 25 seconds.

**Figure 1:**
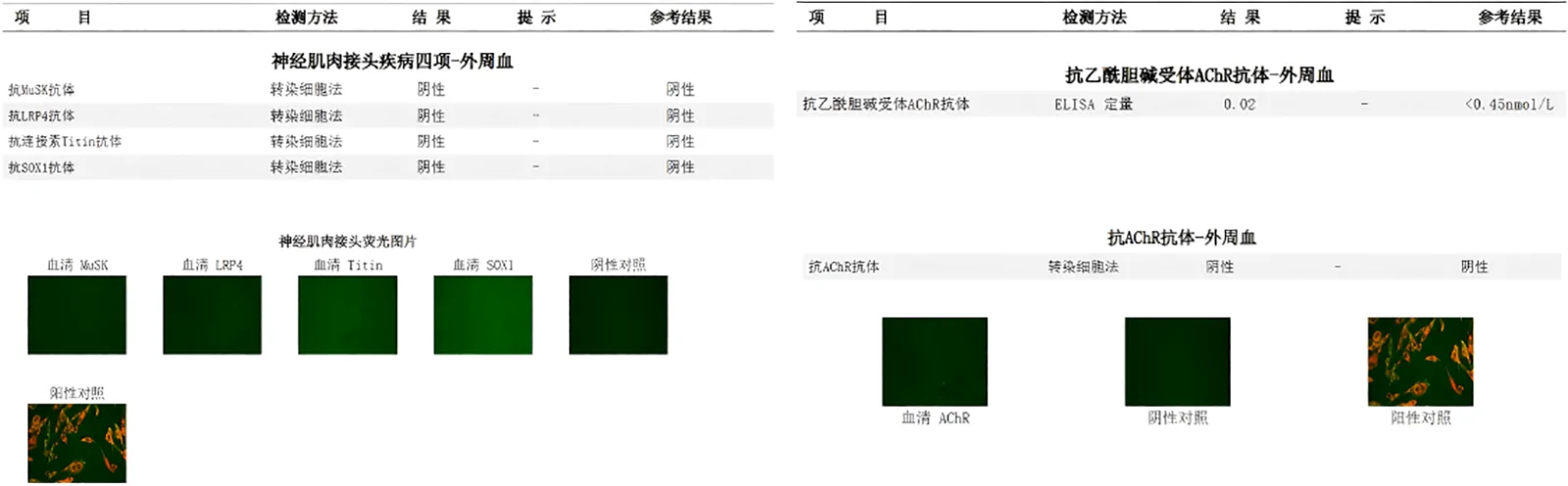
Clinical report summary showing ELISA and immunofluorescence‑based antibody screening results for multiple myasthenia gravis biomarkers.

Laboratory examination: complete blood count: lymphocyte percentage, 15.60%; Autoimmunity: CD3+CD4+% 25.40%, CD3+CD4+/CD8+ ratio 0.18, CD3+CD4+CD103+% 8.50, CD4+ T helper absolute count 399.32, T+B+NK absolute count 1452.64, SSA (52 kDa) positive, SSA (60 kDa) weakly positive, CENP-B weakly positive, AMA-M2 positive. MG-related antibodies: anti-AChR antibody (-), anti-MuSK antibody (-), and anti-LRP4 antibody (-) (**Figure 1**). Chest CT revealed thymic involution insufficiency and a high-density shadow in the mediastinum, suggestive of a pericardial cystic lesion with high protein content. The neostigmine test was positive. 5.0 Hz repetitive nerve stimulation showed: on the right facial nerve, compared with the first stimulus, the amplitude decrement recorded at the orbicularis oculi muscle at the fourth stimulus was 23%.

**Figure 2:**
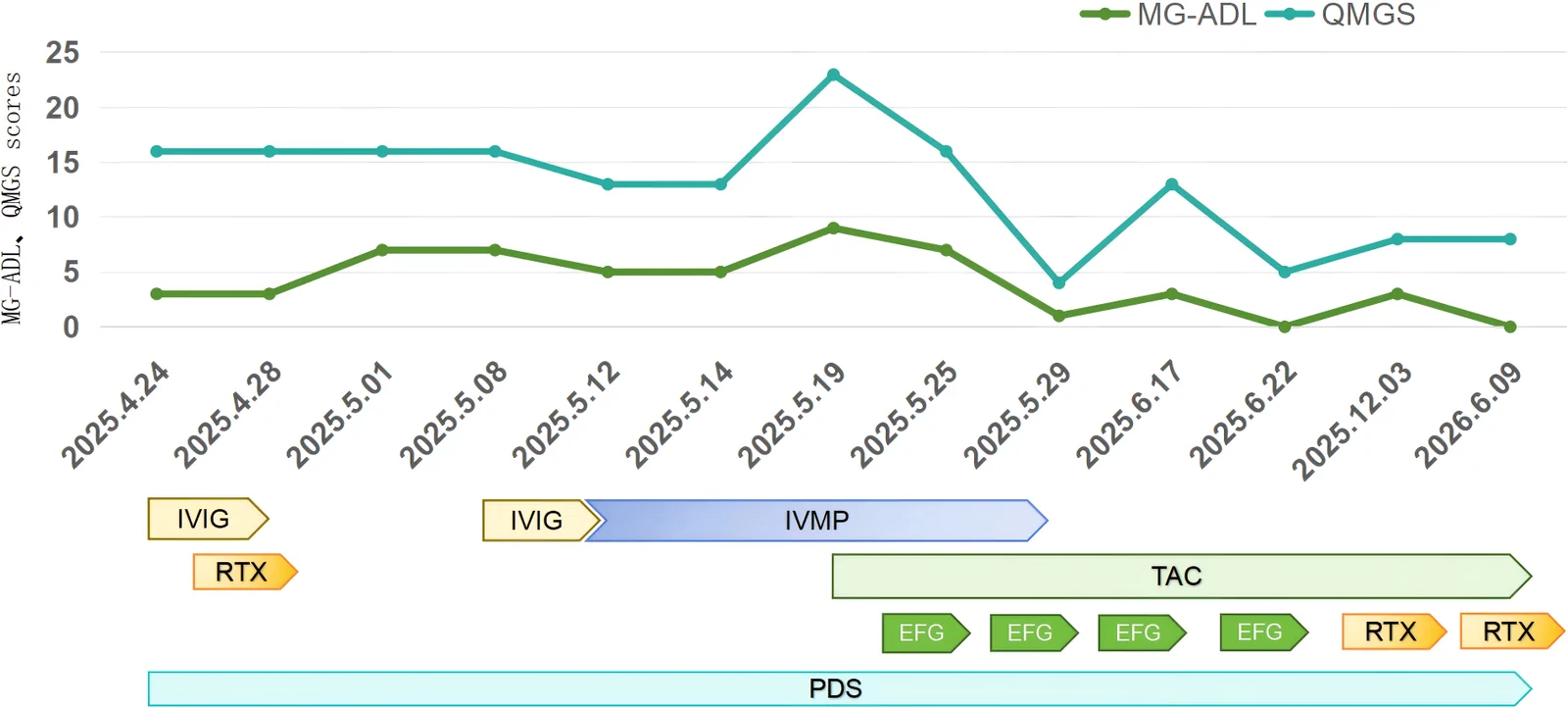
The line graph illustrates the changes in MG-ADL and QMG scores throughout the patient's treatment course. IVIG denotes Intravenous Human Immunoglobulin. RTX refers to Rituximab. INMP stands for Injection Methylprednisolone Sodium Succinate. TAC indicates Tacrolimus. EFG represents Efgartigimod. PDS signifies Pyridostigmine Bromide.

**Figure 3:**
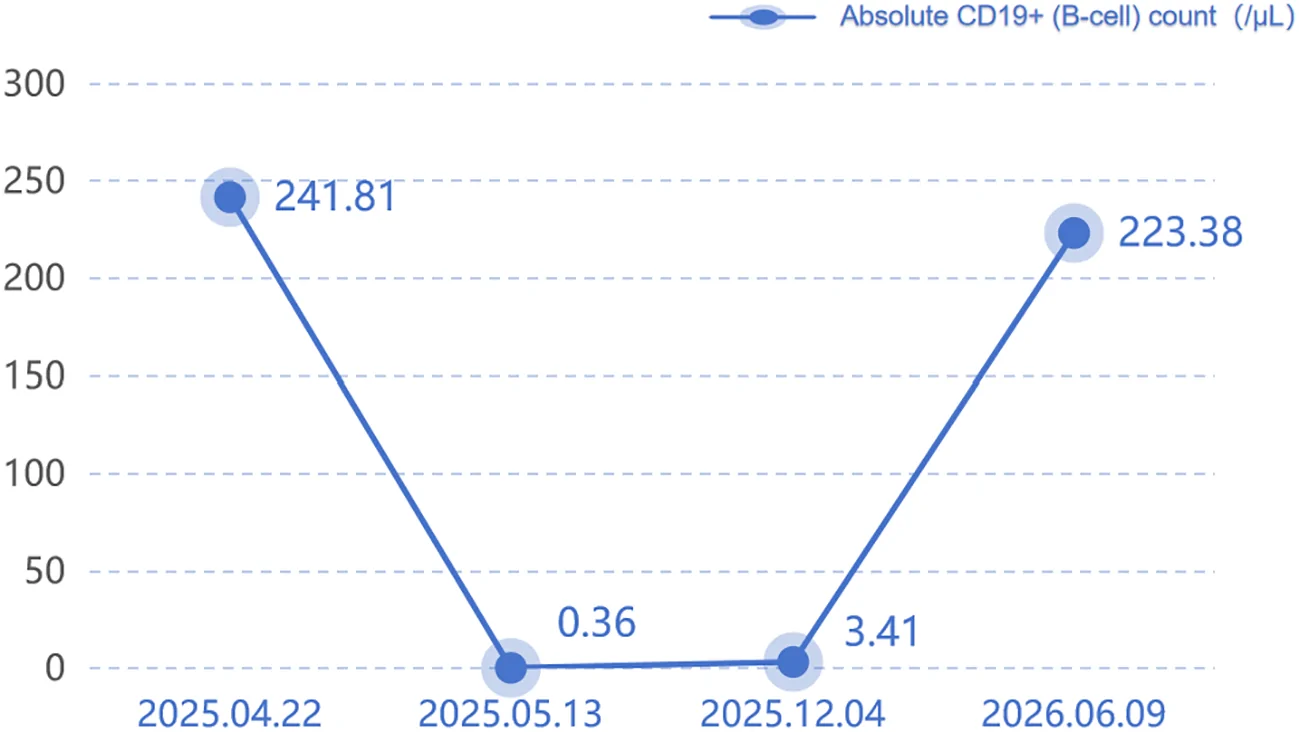
Absolute CD19+ B-cell counts across different treatment stages.

The patient was diagnosed with tSNMG. Initially, pyridostigmine 60 mg orally four times daily was administered, and after 5 days of continuous intravenous infusion of human immunoglobulin 20 g, symptoms still failed to achieve satisfactory control, after informed consent, rituximab (100 mg on day 1, 500 mg intravenous infusion on day 2) was given for targeted therapy. Subsequently, another course of human immunoglobulin 20 g continuous intravenous infusion for 5 days was administered, along with pulse methylprednisolone sodium succinate therapy (500 mg × 5 d, 240 mg × 3 d, 120 mg × 3 d, 40 mg × 3 d). During the early phase of corticosteroid treatment, the patient experienced transient worsening of symptoms, with severe limb weakness and extreme fatigue, MG-ADL score of 9, and QMGS score as high as 23. Tacrolimus 1 mg orally once daily was started for immunosuppression, but no improvement was observed. After further discussion, one cycle of efgartigimod (400 mg × 4) was administered, resulting in significant symptom relief, with MG-ADL decreasing to 0 and QMGS score dropping by 17 points (**Figure 2**). Thereafter, the patient received rituximab maintenance therapy approximately every six months (100 mg × 1, 200 mg × 1). The absolute CD19 (B) cell count decreased from 241.81/μL to 0.36/μL, and at the six-month follow-up remained at 3.41/μL (**Figure 3**). At one-year follow-up, the patient’s MG-ADL score remained 0 (**Figure 2**).

## Discussion

tSNMG is a highly heterogeneous and particularly challenging subtype of myasthenia gravis, characterized by seronegativity for the three classic MG-associated antibodies—anti-acetylcholine receptor (AChR), anti-muscle-specific kinase (MuSK), and anti-low-density lipoprotein receptor-related protein 4 (LRP4) (3).Due to the lack of specific serological markers, clinical diagnosis is extremely challenging and can easily be confused with other neuromuscular disorders such as Lambert-Eaton myasthenic syndrome and polymyositis. tSNMG is associated with a lower treatment response rate and a higher risk of failure than seropositive MG, and the refractory form is more aggressive (2). Its potentially fatal exacerbations can lead to recurrent hospitalizations, severely impairing the patient’s quality of life and imposing a heavy personal and societal healthcare economic burden. The patient presented with generalized fatigue, ocular and limb muscle weakness as the main manifestations. A positive neostigmine test, abnormal electromyography, and myasthenia gravis rating scales, together with negative results for classic MG-related antibodies, confirmed the diagnosis of tSNMG.

tSNMG is not simply ‘antibody-negative’ MG; it may be associated with undiscovered autoantibodies, significant downregulation of lncRNA FAM225A, and dual T/B-cell immune dysregulation (4) In this regard, the patient’s chest CT revealed a benign mediastinal lesion, which indirectly interfered with thymic immune function, induced T/B cell-mediated immune dysregulation, and disrupted self-tolerance. Although it lacked the direct pathogenic effect of a typical thymoma, it mildly stimulated the thymus, leading to abnormal immune regulation, primarily via cell-mediated immune pathways. Even in the absence of definite MG-related antibodies, it could trigger neuromuscular junction transmission disorders, ultimately manifesting as myasthenic symptoms. This was highly consistent with the patient’s concurrent T cell subset abnormalities and the positivity of multiple autoantibodies (SSA [52 kD, 60 kD], CENP-B, AMA-M2). It manifested as a poor response to multiple immunosuppressive therapies, intermittent need for IVIG, or inability to taper corticosteroid dosage without relapse. Traditional broad-spectrum immunosuppressants cannot specifically block this core pathway and frequently cause systemic multi-organ adverse effects. Notably, this patient developed severe transient exacerbation of symptoms and gastric discomfort during glucocorticoid pulse therapy. For refractory tSNMG that is unresponsive to conventional treatment, precise and effective targeted therapeutic strategies are urgently needed in clinical practice. The sequential targeted therapy combining rituximab and efgartigimod, which achieves B-cell depletion and accelerates IgG degradation, closely aligns with the immunopathological features of tSNMG, thereby achieving effective disease control in this refractory MG subtype (5).

In the field of neurology, treating myasthenia gravis presents several challenges. One of these challenges is finding effective therapies that target the underlying mechanisms of the disease while minimizing side effects. Sequential rituximab and efgartigimod has become an attractive option. Rituximab is a monoclonal antibody targeting the CD20 antigen present on 95% of mature B cells. It acts by initiating complement-dependent cytolysis or antibody-dependent cell-mediated cytotoxicity, thereby suppressing autoantibody production at the source, blocking the abnormal autoimmune cascade, and providing long-term regulation of immune homeostasis. In multiple previous observational studies, rituximab has been shown to be effective for new-onset refractory MG (6), A 2019 retrospective study included 4 seronegative patients, of whom 3 showed improvement on the MGFA-PIS scale after receiving rituximab treatment (7), a 2021 meta-analysis included 20 seronegative patients treated with rituximab, of whom 40% met or exceeded minimal manifestation status (8). Furthermore, existing evidence suggests that both low-dose and standard-dose rituximab are effective and comparable in treating myasthenia gravis (9).

Efgartigimod is an Fc fragment of human IgG1 antibody and is the first FcRn inhibitor to receive regulatory approval in multiple countries for the treatment of AChR antibody-positive MG. Efgartigimod has demonstrated robust efficacy in improving MG symptoms with a favorable safety profile. Relevant studies indicate that the novel selective IgG-lowering mechanism of efgartigimod via FcRn receptor blockade is well tolerated in seronegative MG patients. After efgartigimod treatment, the MGII score decreased by 11.92 points (p < 0.01), and the MG-ADL score also improved (10)., another study of 6 seronegative MG patients showed that after efgartigimod treatment, MG-ADL scores improved by at least 5 points in 58% of treatment cycles (11). In the pivotal ADAPT study, assessment of daily living activities in patients with myasthenia gravis showed that seronegative MG patients had response rates similar to those of AChRAb-positive patients (12), Chinese clinical guidelines indicate that (13), regardless of AChR antibody status, clinically meaningful improvements in MG-ADL and QMG scores are observed, and long-term treatment has not been associated with an increased risk of serious adverse events. In this case early administration of rituximab cleared pathogenic B cells, thereby suppressing the autoimmune response at its source. Given rituximab’s delayed onset of action and the adverse effects of glucocorticoids in this patient, efgartigimod, with its rapid onset, provided a treatment alternative that was both timely and targeted. This patient showed a favorable response to efgartigimod. It is postulated that seronegative individuals may still carry pathogenic antibodies, which may differ in quantity and affinity from those in other patient subgroups. Efgartigimod may accelerate the clearance of such antibodies, inhibit their recycling *in vivo*, and thereby reduce these pathogenic antibodies, effectively interfering with the disease course (14).

Although this patient experienced a good response and no complications, this is a single case report. Large-scale studies are required to confirm the efficacy and safety of sequential treatment. The patient received only one cycle of efgartigimod induction therapy to achieve rapid symptom control, while long-term maintenance relied on rituximab (15). Mechanistically, efgartigimod may impair the efficacy of rituximab; given the patient’s symptom fluctuations and specific clinical needs, this personalized treatment approach was adopted. Follow-up results indicated that this regimen still effectively eliminated B cells in the body. Notably, rituximab imposes a heavy financial burden on MG patients; after obtaining informed consent, a slightly lower-than-standard dose of rituximab was selected and also yielded good efficacy. This case report indicates that sequential efgartigimod and rituximab treatment can improve the quality of life of patients with myasthenia and achieve effective disease control.

## Conclusion

In a triple-seronegative MG case, efgartigimod combined with rituximab achieved effective disease control. As this patient subgroup is typically excluded from randomized controlled trials, caution should be exercised regarding this indication. Nevertheless, these findings provide crucial early insights for challenging situations with limited treatment options in clinical settings. More evidence from large registries or randomized controlled trials specifically targeting seronegative MG is needed.

## Data Availability

The original contributions presented in the study are included in the article/supplementary material. Further inquiries can be directed to the corresponding author/s.
